# A cross-sectional study of risk factors associated with hypertension in heart and kidney disease patients in Sana'a, Yemen

**DOI:** 10.3389/fcvm.2025.1621750

**Published:** 2025-10-24

**Authors:** Hussein Mussa Muafa, Malika Abdu Balkam

**Affiliations:** ^1^ Department of Internal Medicine, 21 September University for Medical and Applied Sciences, Sana'a, Yemen; ^2^ Department of Internal Medicine, Ibn Mussa International Hospital, Al Hodaidah, Yemen; ^3^ Department of Medicine, College of Medicine and Health Sciences, Sana’a University, Sana'a, Yemen

**Keywords:** hypertension, heart disease, kidney disease, risk factors, Yemen, Qat, smoking, medication adherence

## Abstract

**Background:**

Hypertension is a major contributor to cardiovascular and renal disease worldwide, particularly in low-resource settings such as Yemen.

**Objective:**

To identify demographic, clinical, and lifestyle-related factors associated with hypertension among heart disease (HD) and kidney disease (KD) patients in Sana'a.

**Methods:**

A cross-sectional study was conducted on 300 patients (200 men, 100 women; aged 35–70 years) between March and August 2024. Data on age, sex, weight, smoking, Qat chewing, diabetes, and antihypertensive medication adherence were collected. Blood pressure was measured using a standardized sphygmomanometer. Logistic regression was used to calculate odds ratios (ORs) and 95% confidence intervals (CIs).

**Results:**

Hypertension was significantly associated with age (OR: 1.08; 95% CI: 1.02–1.11), obesity (OR: 2.42; 95% CI: 1.23–4.75), smoking (OR: 1.88; 95% CI: 1.05–3.35), type 2 diabetes (OR: 2.87; 95% CI: 1.56–4.55), and irregular medication use (OR: 3.21; 95% CI: 1.45–7.11). All participants reported Qat chewing (100%).

**Conclusion:**

Age, obesity, smoking, diabetes, and poor medication adherence are major predictors of hypertension in Yemeni HD and KD patients. The universal irregularity in medication use highlights systemic healthcare gaps. Strategies to improve adherence, promote lifestyle modification, and strengthen healthcare systems are urgently needed.

## 1 Introduction

Hypertension is one of the most significant risk factors for cardiovascular and renal diseases. In LMICs, including Yemen, limited healthcare infrastructure exacerbates the burden of hypertension. The disease accelerates progression to myocardial infarction, stroke, and end-stage kidney failure, particularly in individuals already suffering from HD or KD (1, 2).

Regional studies have identified obesity, smoking, diabetes, and poor adherence to therapy as key drivers of hypertension (1, 2). In Yemen, widespread Qat chewing further complicates the risk profile, though its precise contribution remains unclear (3).

This study investigates demographic, clinical, and behavioral risk factors for hypertension among Yemeni HD and KD patients (1, 3–5), aiming to inform culturally appropriate prevention and management strategies (6).

## 2 Methods

### 2.1 Study design and setting

A cross-sectional study was carried out in hospitals and outpatient clinics in Sana'a, Yemen, between March and August 2024.

### 2.2 Study population

300 patients with HD, KD, or both were recruited via convenience sampling.

**Inclusion criteria:**

• Age 35–70 years
• Diagnosed with HD or KD
• Provided informed consent

### 2.3 Data collection

Structured interviews and medical records were used.

• **Demographics:** age, sex, marital status
• **Clinical data:** weight, diabetes, blood pressure
• **Lifestyle:** smoking, Qat chewing
• **Medication adherence:** self-reported regular vs. irregular

**Blood pressure measurement:** Conducted with a calibrated sphygmomanometer, two readings averaged after 5 min rest. Hypertension defined as ≥130/80 mmHg (ACC/AHA 2017).

### 2.4 Statistical analysis

SPSS v25 was used. Logistic regression assessed associations between risk factors and hypertension. Significance: *p* < 0.05.

## 3 Results

### 3.1 Baseline characteristics

Table 1.

**Table 1:** Baseline characteristics of participants (*N* = 300).

Variable	Frequency (*n*)	Percentage (%)
Age (35–70 years)	300	100.0
Male	200	66.7
Female	100	33.3
Married	300	100.0
Weight 70–90 kg	250	83.3
Weight 90–120 kg (Obese)	50	16.7
Qat chewing	300	100.0
Smoking	250	83.3
Type 2 diabetes mellitus	150	50.0
Irregular antihypertensive use	300	100.0

### 3.2 Logistic regression analysis

Table 2.

**Table 2:** Logistic regression analysis of risk factors for hypertension.

Risk factor	Odds ratio (OR)	95% CI	*p*-value
Male gender	1.35	0.92–1.99	0.11
Age (per year increase)	1.08	1.02–1.11	0.004*
Obesity (90–120 kg)	2.42	1.23–4.75	0.01*
Smoking	1.88	1.05–3.35	0.03*
Type 2 diabetes mellitus	2.87	1.56–4.55	0.001*
Irregular medication use	3.21	1.45–7.11	0.004*

* Significant at *p* < 0.05.

## 4 Discussion

This study demonstrated that age, obesity, smoking, diabetes, and irregular antihypertensive drug use were significant predictors of hypertension (7–9). The 100% prevalence of irregular drug adherence reflects systemic issues in the Yemeni healthcare system (1, 2).

**Key findings:**

• **Age:** Vascular stiffening and cumulative exposure to risk factors explain the increased risk.
• **Obesity:** Strong predictor of hypertension via increased cardiac output and vascular resistance.
• **Smoking:** Nicotine contributes to endothelial dysfunction and sympathetic activation.
• **Diabetes:** Insulin resistance and arterial stiffness exacerbate BP rise.
• **Medication adherence:** The strongest predictor, highlighting systemic deficiencies (9, 10).

**Qat chewing:** Could not be analyzed statistically due to uniform exposure but remains a critical cultural factor requiring future comparative studies.

**Strengths:** First study in Yemen on this specific patient population; robust statistical analysis.

**Limitations:** Cross-sectional design; convenience sampling; lack of control group; inability to analyze Qat chewing due to uniformity.

**Implications:**

• Improve medication access and adherence programs (10).
• Strengthen public education on lifestyle modification.
• Develop system-level policies for better hypertension management.

## 5 Conclusion

Hypertension among Yemeni HD and KD patients is significantly associated with age, obesity, smoking, diabetes, and poor medication adherence (11, 12). Addressing these factors through systemic reforms and culturally tailored interventions is essential to reduce the growing cardiovascular and renal burden in Yemen.

## Data Availability

The original contributions presented in the study are included in the article/Supplementary Material, further inquiries can be directed to the corresponding author.
